# Some Theories as to the Causation of Cancer

**Published:** 1903-04

**Authors:** Greer Baughman

**Affiliations:** Richmond, Va., Lecturer on Hygiene; Demonstrator of Pathology and Physiology, Medical College of Virginia; Member of State Medical Society, Tristate Medical Society of Virginia, North and South Carolina, American Medical Association, Southern Surgical and Gynecological Society, etc.


ATLANTA

### Journal-Record of Medicine.

**Successor to Atlanta Medical and Surgical Journal, Established 1855, and Southern Medical Record, Established 1870.**

VOL. V. APRIL, 1903. No. 1.

BERNARD WOLFF, M.D., EDITOR, Nos. 319–20 Prudential.

Published Monthly.

M. B. HUTCHINS, M.D., BUSINESS MANAGER, No. 64 Marietta St.

### *ORIGINAL COMMUNICATIONS.*

### SOME THEORIES AS TO THE CAUSATION OF CANCER.*

BY GREER BAUGHMAN, M.D., RICHMOND, VA.,

Lecturer on Hygiene; Demonstrator of Pathology and Physiology, Medical College of Virginia; Member of State Medical Society, Tristate Medical Society of Virginia, North and South Carolina, American Medical Association, Southern Surgical and Gynecological Society, etc.

Such an intimate acquaintance has the world had with malignant epithelial neoplasms that a familiar household name has been given them. "Karkinos," or crab, was the name used by Galen to signify such sores of the breast as were characterized by malignancy, by tendency to return after being removed, and by rapidity of growth. The Germans call cancer "krebs," which means crab. Sanskrit "Karkata" and "Karkataka," used in the same sense, meaning crab, applied to cancers. In Hindustani "Kark" (crab)

*Read before the Richmond Academy of Medicine and Surgery, February 24, 1903.

is used in the same sense. All of these people had in their mind a description of the disease as well as a name for it.

From the examinations of Bischat, Lobstein and others, Læennec supported the idea that cancers were heterologous, in other words, that they were different from the cells of the body. J. Müller locked horns with him, for he demonstrated that the cancer cells were identical with the normal, physiologically acting cells of the body. It was the brilliant and many-sided Virchow, however, who finally established, beyond contradiction that the cancer cell, as well as all tumor cells, were identical with the cells of the body, and who gave a new meaning to homologous and heterologous—making homologous mean tumors that grow among similar, and heterologous mean tumors that grow among dissimilar cells from themselves. But he defended the idea, and for his whole life believed that cancers were of connective tissue origin.

This theory of his was energetically and bitterly opposed by such men as Thiersch and Waldeyer. Virchow's reputation was so well known and his defense was so masterly, that only in the last few years have scientists given up the connective tissue origin theory. There are to-day but few, if any, pathologists who do not believe that cancers are of epithelial origin.

Of the many opinions as to the causation of cancer that have been advanced since the time of Galen, the ones that have had the most facts to support them are: 1, The parasite; 2, Thiersch's theory; 3, Cohnheim's; 4, Hauseman's; 5, Ribbert's; 6, Wakefield's; and 7, the bacteriological.

Virchow overturned the parasite idea when he established the fact that all cells of tumors find their prototype in the human body. Before discussing the other theories as to exciting causes, it might be well to discuss briefly the usual place of finding cancers and to mention some of the predisposing causes.

It has been noted after many years of investigation that cancers select certain organs for their primary growth in order of their frequency: the uterus (vaginal portion); outer skin (lip, ear, eyelid, cheek, extremities); female breast (much seldomer the male breast); stomach (pyloric end, seldomer the cardiac, and rarely the fundus); rectum; esophagus; ovaries; outer genitalia; penis; scrotum; clitoris; labium majus; vagina; prostate and bladder; testicle and epididymis; abdominal viscera (particularly the pancreatic head); small intestine; gall-bladder; gall-ducts; liver; thyroid gland; kidney; bronchi; lungs; tube; ureter; vesiculæ seminales; ventricles of brain; dermoid cysts; bone, etc.

Almost every patient suffering from a cancer, particularly when the cancer is superficial, will give the history of some injury to the affected part. So prominent is this fact that at one time it was generally believed that direct violence was the cause of cancer. The causative effect of age will be discussed later. Sex as a predisposing cause cannot be elaborated any more than mentioning the facts that men are more subject to injury, if that be a cause of cancer, while women bear and suckle the babies. Race seems to have something to do with the appearance of this dread pathological lesion. Hygienic surroundings and contact, no doubt, have their bearing, and a run-down condition certainly does.

Carcinomas occur usually in old people. Thiersch used this fact as the foundation upon which he built his theory. He believed that the initiative for cancerous growth came from the epithelium, but that a lessened resistance on the part of the connective tissue stroma helped. He theorized that this lessened resistance was due to a poor blood supply, which would be the case of those suffering from sclerotic arteries and the like; consequently cancers were found almost exclusively among the older people.

Cohnheim explained the tendency on the part of the cancer cells to proliferate as due to the embryological nature of their cells. He believed that all tumors originated in the same way by assuming that more cells were manufactured then were necessary for building the organs or tissues of the embryo; so long as the normal physiological cells were actively doing their allotted duty and were healthy, the embryological excess cells had no chance to grow. Just as soon as the physiological cells ceased to multiply to increase the size of the organ or tissues, but more particularly when the tissues began to degenerate from age, the embryological excess cells commenced to assert themselves, and being embryological in character, their growth was rapid. From Cohnheim's theory we would naturally expect to find tumors either about puberty or in old age, or whenever the body gets into a run-down condition. This theory was substantiated by a series of experiments conducted by Leopold, who attempted to grow a piece of cartilage taken from a full-grown guinea-pig in the abdomen of another. The experiment failed. But when a piece of cartilage from an embryological guinea-pig was used an enchondroma was grown.

Hauseman, of Berlin, who knows more about tumors than any living man, in my opinion, explains the origin of tumors by the theory that certain epithelium undergoes a retrograde metamorphosis, taking on the growth characteristic of the embryological cells, and losing in part, if not wholly, their secretive and other properties. They, in this way, have gained growing powers at the loss of their differentiating and functioning properties. This is known as the "anaplasia of cells."

Ribbert assumes that the first changes in beginning carcinomas take place in the connective tissue, which, for some such reason as chronic inflammation or hypertrophy, cuts off certain epithelium from its accustomed associates. These cells, not being in their usual place and having now no especial function to perform, grow to be tumors.

A more recent theory of Wakefield might be mentioned at this point. He believes that gelatiniform tissues, as observed in neoplasms and elsewhere, invariably represent a stage of degeneration of tissues once healthy, the said degeneration bearing no relation whatever to the embryology of the tissues. The conditions responsible for the increase in the involved tissue is a retardation of catabolism. He also believes that tissues are subject to tumor formation in inverse ratio to their catabolic digestibility, this digestibility being aided by an alkaline medium.

To illustrate these theories in a practical way, Cohnheim's would be a man who was born with a desire to steal; this desire was kept in subjection for fear of punishment by laws formulated by his neighbors for their mutual protection; opportunity presenting in such a way that he thinks he will not be caught, he steals, the habit then grows upon him until presently he is found out, punished, and looked upon by his neighbors as an outcast—a tumor.

Hauseman's theory is the citizen that allows his passion to take away his energy and desire to work; he degenerates into the bum found at every almshouse, who gets fat at the expense of the community.

Ribbert's idea of cancer causation might be likened to the cancer quack, who began legitimate practice with bright prospects and many friends, but who, because of the desire to get rich quickly, cut himself off from the honorable practitioners, by this very cutting-off losing the desire to uphold the dignity of the profession, to further the good of his fellow doctors and of the community as a whole, and expended all his energies in amassing a fortune, preying, as does the tumor, on the organism of the commonwealth.

Wakefield's theory is the man that accumulates a fortune by failure to pay his debts. All of us could be tumors of this kind in a short time if we could only get credit.

Many different bacteria as causes of cancer have been described; with none of these except the blastomycetes of Leopold have I any personal knowledge, so, as the time is limited, I shall call your attention to these alone.

Geheimrath Leopold, of the Frauen Klinik, Dresden, along with his assistant, Dr. Rosenthal, began investigations to ascertain if there was a causative bacterium for cancer early in 1894; not until 1900 did he read his paper before the Medical Congress at Paris, publishing his complete investigations to the world.

The material examined was taken from body-warm cancers, removed from various parts of the body; scrapings from the innermost portions were taken with asceptic precautions. These scrapings were examined upon an especially constructed warm microscope stage in sterile beef tea, normal salt solution, or blood serum. Among fatty degenerate cancer cells were found particles with double contour, some of which were filled with yellow-pointed or long-drawn-out particles, highly refractive. These particles seemed to have a peculiar motion; all did not move at once, the motion beginning at the periphery. After they had been moving some time, suddenly a particle pushed through the covering membrane of the cell and the others followed, but no lesion of the cell-wall was to be seen. Other particles formed, and this cycle of procedure would continue for hours. It was not necessary to keep the temperature constant for this process to take place.

In 1898 Leopold reported that he had observed a hanging drop made of freshly prepared carcinomatous tissue in sterile bouillon for two hundred consecutive days.

In 1900 the same observer showed pure cultures of blastomycetes obtained from human cancers; likewise tumors in animals, which were produced by pure cultures of these same blastomycetes obtained from human cancers. The blastomycetes obtained from the tumors of the animals were identical in appearance, in staining peculiarities, and in growth with those obtained from human. To make the circle complete the blastomycetes derived from the animal cancers must be injected into and produce tumors in other animals or in man, which shall likewise contain blastomycetes identical with the others.

The procedure of growing these blastomycetes was first to find them in the sterile bouillon, then aseptically and with care to infect a tube of neutral gelatine. In this tube there was a mixed infection. The second contained a few other germs. The fourth tube gave a pure culture of blastomycetes. These blastomycetes stain with the usual coloring fluids. Hematoxylin and fuchsin or eosin can be used with best results.

Many animals were infected with pieces of fresh tumors. A white rat was infected with a piece from an ovarian cancer; after sixty-one days the rat died of numerous tumors. The microscopical diagnosis was adenosarcoma with many blastomycetes. In the case of a guinea-pig infected with a cervical cancer, the liver was covered with epithelial tumors and contained blastomycetes. A rat injected into the testicle with a pure culture of blastomycetes derived from an ovarian cancer, after 195 days died, filled with numerous tumors. These proved to be round and giant-celled sarcomas with numerous blastomycetes.

To recapitulate, Leopold was able: 1, to see blastomycetes in scrapings made from body-warm cancers and to study them; 2, to make pure culture; 3, to inject a pure culture into the testicle of a rat, that died of tumors in which were found numerous blastomycetes; 4, to make pure culture of the blastomycetes found in the tumors of the rat.

BIBLIOGRAPHY.

Cohnheim—Allgemeine Pathologie, 1882.

Birch-Hirschfeld—Patholog. Anatomie, 1897.

Delafield and Prudden—Handbook of Pathological Anatomy and Histology, 1901.

Proceedings State Society of Virginia—Past, Present and Future of Cancer. Stuart McGuire, 1901.

*American Medicine*, November 22 and 29, 1902—The Pathology of Catabolism in Relation to the Etiology and Pathology of Cancer and Allied States. Homer Wakefield.

*Medical News*, September 20, 1902—The Present Status of Cancer; its Etiology and Pathology. The Value of Laboratory Research. Edward N. Liell.

*Archiv für Gynecologie*, Band 61, Heft 1, 1900—Untersuchungen zur Aetiologie des Carcinoms und über die pathologenen Blastomyceten. Geheimrath Leopold.

Dr. Hugo Ribbert—Lehrbuch der Pathologischen. Histologie, 1896.

Dr. David Hauseman—Die Microscopische Diagnose der Bosartigen Geschwülste, 1897.

12 N. Second Street.

